# News and Events of the Week

**Published:** 1910-07-30

**Authors:** 


### News and Events of the Week.

Dr. Edward C. Seaton, Medical Officer of Health for Surrey, is resigning his post. Dr. Seaton's work in public health has covered a period of nearly forty years, twenty of which he has spent under the Surrey County Council.

His Majesty the King has graciously consented to become Patron of the Royal Sanitary Institute, which was founded in 1876 and is carrying on a large work in teaching and examining in hygiene and sanitary science, both in the United Kingdom and in other parts of the Empire. It maintains in London a permanent museum of sanitary appliances, open free to the public, and has a membership of 4,000.

At the graduation ceremony for the Faculty of Medicine of the Edinburgh University Professor Sir Thomas Fraser asked the Vice-Chancellor, Sir William Turner, to present the Cameron Prize in Therapeutics to Professor August Bier, Professor of Surgery in Berlin University, "for the additions to practical therapeutics embodied in his work on spinal anæsthesia and on hyperæmia as a therapeutic agent."

The following sessional awards have been made by the Medical Faculty of the Edinburgh University: Thesis Gold Medals.—George C. Low, M.A., M.D., William K. MacDonald, M.D., William M'D. Scott, M.D., William H. Welsh, M.D., and James Young, M.D. Syme Surgical Fellowship.—William K. MacDonald, M.D., Ch.B. Ellis Prize in Physiology.—Robert A. Krause, M.B., Ch.B. Gunning Victoria Jubilee Prize in Surgery.—John Fraser, M.B., Ch.M. Ander Henry Prize in Botany.—Bertha Chandler, M.A., B.Sc. Ettles Scholarship.—Francis Gordon Bell, M.B., Ch.B. Allan Fellowship in Clinical Medicine and Clinical Surgery.—John Drummond, M.B., Ch.B. Conan Doyle Prize.—Charles J. G. Bourhill, M.B., Ch.B. Annandale Gold Medal in Clinical Surgery.—J. J. Tough, M.B., Ch.B. Buchanan Scholarship in Gynecology.—Matthew R. Drennan, M.A., M.B., Ch.B. James Scott Scholarship in Midwifery.—Keith B. MacGlashan, M.B., Ch.B.

The Romford Garden Suburb at Romford, Essex, the latest expression of the garden city and town-planning movement, was inaugurated on Thursday, July 28, at 1 p.m., when Mr. John Burns laid the foundation-stone of the first house.

At the last meeting of the Council of the Royal College of Surgeons Mr. R. Clement Lucas was elected a member of the Executive Committee of the Imperial Cancer Research Fund. Mr. Stephen Mayou, F.R.C.S., has consented to undertake the revision of the museum collection of the ophthalmological specimens, and Mr. Arthur H. Cheatle, F.R.C.S., will undertake the revision of the ear specimens. The president reported that the term of office of Mr. A. S. Underwood on the Board of Examiners in Dental Surgery would expire on October 12 next, and that the vacancy thus occasioned would be filled up at the quarterly meeting of the council in that month.

On Tuesday evening last Mr. H. T. Butlin, the President-elect of the B.M.A., entertained to dinner at the Hotel Cecil about 400 representative medical men from all parts of the country, the overseas Dominions, the Continent of Europe, and America now assembled in London for the annual meeting of the association. Lord Ilkeston (Sir B. W. Foster) proposed Mr. Butlin's health. He said that during his long memory of the association there had been few men more capable of filling the office of president with grace, with eloquence, with dignity, and with business-like aptitude, and he was sure that they all wished Mr. Butlin long life and a successful and happy year of office. Mr. Butlin, in reply, said that he hoped to be able to discharge his duties as president to the satisfaction of the association. Lord Ilkeston, he added, had done more than any other living man for the British Medical Association and for the public measures which had been brought before Parliament by the association and by the profession. There was no one in the profession better fitted to represent them in the House of Lords than Lord Ilkeston.

To-day parties of medical men will visit Lord Mayor Treloar's Cripples' Home and the Hot Springs at Bath by special invitation.

Members of the British Medical Association took advantage of the invitation of the Royal Sanitary Institute to inspect the buildings of the Institute and the Parkes Museum on Wednesday last.

Mr. Butlin delivered his presidential address to the British Medical Association on Tuesday evening last in St. James's Hall. We hope to deal with this and other addresses delivered before the Association on a future occasion.

Mr. Pearce Gould presided at the annual breakfast of the National Temperance League on Thursday morning last. The breakfast was held at the Imperial Institute, and was well attended. During the last few years the membership of the League has steadily increased.

Having failed to get the necessary assistance from the cottage hospital to deal with the many cases of defective eyesight in children attending the Acton Elementary Schools, the local education committee has appointed Dr. Grace Banham as lady medical oculist for twelve months.

The Medico-Psychological Society held its annual meeting in the Hall of the Royal College of Physicians, Edinburgh, last week. Professor W. Bevan-Lewis, the retiring President, was in the chair. Dr. John Macpherson, of Edinburgh, was elected President; Dr. Thomas Drapes, of Dublin, President-elect; and Dr. C. Hubert Bond, Secretary. Dr. Macpherson delivered an address on "The Conceptions of Insanity and their Practical Results."

At the representative meeting of the B.M.A. on Monday last it was stated that the Poor-Law Committee has prepared a scheme for a centrally controlled public medical service. The recommendations contained in the scheme report will have to be considered by the divisions of the association before final adoption. Provision is made for the organisation by the British Medical Association of a national public medical service, which should be administered by the local medical profession, in so far as matters of professional import are concerned, and by joint committees of medical and non-medical members, in so far as non-professional matters are concerned. A discussion took place on the nature of the contract which is to be made between the medical attendant on the one side and the service on the other. A number of speeches were delivered by members who had studied the workings of the State insurances in Germany and the conditions of contract practice in this country. With regard to the question of remuneration of the medical attendant, the Medico-Political Committee advised as follows: "That the payments of all beneficiaries in the district of one local committee be paid into a local common fund, and that after payment of locally agreed expenses the balance be distributed periodically among the medical officers of that area according to such principle as may be agreed upon among themselves—*e.g.* either by a payment per head in respect of each beneficiary who is for the time being on the list of each doctor, or by payment of fees in respect of attendance actually given by each doctor, or by such combination of these methods or such other method as may be locally approved." The recommendation of the committee was approved.

